# Inner Correspondence and Peacefulness with Practices among Participants in Eurythmy Therapy and Yoga: A Validation Study

**DOI:** 10.1155/2011/329023

**Published:** 2010-09-07

**Authors:** Arndt Büssing, Friedrich Edelhäuser, Annette Weisskircher, Judith M. Fouladbakhsh, Peter Heusser

**Affiliations:** ^1^Center for Integrative Medicine, Chair of Theory in Medicine, Integrative and Anthroposophic Medicine, Faculty of Medicine, University of Witten/Herdecke, Gerhard-Kienle-Weg 4, 58313 Herdecke, Germany; ^2^Integrated Studies of Anthroposophical Medicine, University of Witten/Herdecke, Gerhard-Kienle-Weg 4, 58239 Herdecke, Germany; ^3^Chair of Eurythmy Therapy, Alanus University of Arts and Social Sciences, Villestraße 3, 53347 Alfter, Germany; ^4^College of Nursing, Wayne State University, 5557 Cass Aveune, Detroit, MI 48202, USA

## Abstract

Several mind body medicine interventions require an active participation of the practitioners. We intended to develop a questionnaire to operationalize and measure the “inner correspondence” of individuals practicing Yoga or Eurythmy Therapy. In an anonymous cross-sectional study we enrolled 501 individuals (61% yoga). Exploratory factor analysis (study 1) of the 12-item instrument (Cronbach's alpha = .84) pointed to a 3-factor solution, with one major scale and good internal consistency (alpha = .83) and two minor scales with weak internal consistency. To improve the quality of the main scale, we added 8 new items which were tested in a sample of 135 individuals (study 2: 71% Yoga). Factor analysis confirmed a 12-item single factor (alpha = .95), that is, *Inner Correspondence/Peaceful Harmony with Practices * (ICPH). The scale correlated strongly with mindfulness (FMI; *r* > .50), moderately with life and patient satisfaction (BMLSS; *r* between .32 and .43), and weakly negative with symptom score (VAS; *r* = −.23). In conclusion, the scale ICPH was confirmed as a relevant tool to measure the inner correspondence and feelings of peacefulness with practices. It can be used in clinical studies to assess the efficacy of mind-body practices involving physical movements.

## 1. Introduction

Several complementary and alternative medicine treatments require active participation of the patient, particularly those from mind-body medicine (MBM). One of the core concepts of MBM is that mental, emotional, social, behavioral, and spiritual factors have an impact on patients' health situation, ways of coping, and self-care. Typical mind-body medicine approaches are meditation, relaxation, yoga, tai chi, qi gong, and several more. All of these focus on the unique point of view that it is not the remedy or the therapist that actively heals but rather may induce processes of healing, inner transformation, and so forth—which are active powers of the patients.

One popular representative of the so-called “Whole Medical Systems” with a complete system of theory and practice and a distinct usage of mind-body-based approaches is Anthroposophic Medicine (AM). It was founded in the 1920s by the Austrian philosopher Rudolf Steiner and the Dutch physician Ita Wegman [1]. The core concepts are that the body is not an independent material organism and that health depends on a harmonious relationship between the physical body, vital force, soul, and “spiritual self.” AM therefore intends to address the salutogenetic capacities of the patients and to strengthen their autonomy [2]. One of the important therapeutic strategies in AM is Eurythmy Therapy (EYT, “harmonious rhythm”), introduced by Rudolf Steiner in 1911. This treatment can be described as an active exercise therapy, involving cognitive, emotional, and volitional elements [3]. 

Although there are some similarities with (psychotherapeutic) dance therapy, EYT is a unique movement therapy in which speech movements are transposed into exercises which address the patient's capability for soul expression and strengthen his salutogenetic resources. These specific body movements go along with meditative aspects in terms of guided imagery (inner pictures) and sound. EYT is assumed to have general effects (i.e., improving breathing patterns and posture, strengthening muscle tone, and enhancing physical vitality [2]) and specific therapeutic effects. Moreover, EYT claims to stimulate somatic healing processes through the soulful experience of the respective movements [4]. A recent study by Seifert et al. investigated the effects of EYT on heart rate variability and found stimulation effects with a decrease of low frequency/high frequency ratio, indicating an improved relaxation of healthy individuals [5]. A recent systematic review on the effects of EYT in clinical studies found positive treatment effects with clinically relevant effect sizes in most cases [6], that is, significant improvement of disease and symptom scores in patients with various chronic diseases and in children with anorexia nervosa. EYT could be regarded as a potentially relevant add-on in a complex therapeutic approach which intends to support health and well-being (salutogenesis) although its specific relevance remains to be clarified. For the patients, there is no need to agree with the views of AM, but they are advised to follow the external movements of EYT with guided imagery [6].

A contrasting approach which shares some similarities but also significant differences is Yoga. It refers to traditional disciplines of the Hindu philosophy and involves physical and mental practices. However, there is not one Yoga style but several branches which differ in philosophy and forms of practice (i.e., Raja Yoga, Karma Yoga, Hatha Yoga, etc.). Yoga aims to calm the mind and “self” through different forms of mental development, physical practice in terms of distinct postures (*asanas*), and rhythmic control of breath (*pranayama*); it integrates awareness of breath, relaxation, exercise, and social support, which are key elements to enhance quality of life. A recent review indicated that yoga interventions can result in a short-term reduction of body weight, blood pressure, glucose level, and high cholesterol while only a few studies examined long-term adherence [7]. Other studies have found significant effects of yoga in the treatment of patients with migraine [8] and in patients with irritable bowel syndrome [9]. Brown and Gerbarg provide clinical evidence “for the use of yoga breathing in the treatment of depression, anxiety, post-traumatic stress disorder, and for victims of mass disasters”, because Yoga may induce stress resilience [10]. Another study investigated women perceiving emotional distress, found improvements in perceived stress, depression, anxiety, fatigue, and well-being [11]. In the treatment of cancer patients, yoga practice may assist to manage symptoms such as depression, anxiety, insomnia, pain, and fatigue [12]. Also in patients with major depression (although only a small number of patients were enrolled), yoga intervention significantly reduced depression, anger, anxiety, and neurotic symptoms [13]. Yoga practices may in fact enhance well-being, mood, attention, mental focus, and stress tolerance by an increased parasympathetic drive, calming of stress response systems, neuroendocrine release of hormones, and/or thalamic generators [14, 15]. Khattab et al. [16] reported that—similar to the findings in EYT [5] —yoga may increase the heart rate variability, particularly parameters associated with vagal tone. 

However, interventions such as EYT, Yoga, tai chi, and qi gong. require an active (emotional) engagement of individuals practicing it. One may suggest that the effectiveness of these treatments is dependent on patients' inner involvement, too. The aim of the study was thus the development of a test instrument which operationalizes and measures the “inner correspondence” of individuals engaged in specific mind body interventions such as EYT and Yoga as a prerequisite to address the aforementioned hypothesis in future studies. We assume that this “inner correspondence” will increase with duration of practice and is associated with life satisfaction, and peaceful easiness in response to the practices.

## 2. Methods

### 2.1. Participants

In an anonymous cross-sectional survey, we enrolled 501 individuals attending either individual EYT (39%) or Yoga courses (61%). There was a higher prevalence of women (86%); all further demographic details are given in Table 1.

Individuals provided informed consent to participate by returning the completed questionnaire which did not ask for names, initials, addresses, or clinical details (with the exception of a diagnosis). The cross-sectional survey was approved by the ethical commission of the University of Witten/Herdecke (#63/2009).

### 2.2. Measures

We intended to measure the internal congruence while practicing EYT or yoga exercises (either Yoga Vidya or Hatha Yoga) which involves a mixture of emotional, behavioral, cognitive, and spiritual components. We relied on the motifs mentioned by EYT therapists, representatives of the AM movement and scientists at a Conference on EYT research at the Alanus University of Arts and Social Sciences, Alfter, and focused on the attitudes of consciousness, inner accordance with exercises, and following “inner pictures” (in terms of evoked imagery). The primary 16-item questionnaire was sent to the respective experts and, after marginal modifications in item phrasings, was administered to the study participants.

The questionnaires were provided to EYT therapists in the Communal Hospital Herdecke in Herdecke, the Ita-Wegman Clinic in Arlesheim, the Lahnhöhe Clinic in Lahnstein, and specialized EYT wards in Bovenau, Berlin, Krefeld, Hamburg, Stuttgart, Heidelberg, and other and were also administered during yoga courses in the Department of Mind Body Medicine, Internal and Integrative Medicine at the Essen-Mitte Clinics (Hatha Yoga) and the Yoga Vidya Centers Cologne, Darmstadt, Kelbra, Schwäbisch Gmünd, Schwerte, and Speyer, respectively (Yoga Vidya). All items were scored on a 5-point scale from disagreement to agreement ((1) does not apply at all; (2) does not truly apply; (3) do not know; (4) applies quite a bit; (5) applies very much) and are referred to a 100% level (transformed scale score). Scores >50% indicate higher agreement (positive attitude) while scores <50 indicate disagreement (negative attitude). Four items were recoded because of a negative phrasing (i.e., B8, B10, B14, and B15).

Life satisfaction was measured with the 8-item “Brief Multidimensional Life Satisfaction Scale” (BMLSS; Cronbach's alpha = .87) [17]. The items of the BMLSS refer to intrinsic dimensions (Self, Overall life), social dimensions (Friendships, Family life), external dimension (Work, Where I live), and the perspective dimensions (Financial situation, Future prospects) [17]. Patients' satisfaction was measured with 3 additional items (i.e., health situation, effectiveness of treatment, and own abilities to deal with daily life). Each item was introduced by the phrase: “I would describe my satisfaction with …”, and scored on a 7-point scale from dissatisfaction to satisfaction ((1) Terrible; (2) Unhappy; (3) Mostly dissatisfied; (4) Mixed (about equally satisfied and dissatisfied); (5) Mostly satisfied; (6) Pleased; (7) Delighted). The BMLSS sum score was referred to as a 100% level (Delighted).

The perceived affection of health was measured with a symptom score (visual analogue scale ranging from 0 [none] to 100 [unbearable]).

Mindfulness was measured with the Freiburg Mindfulness Inventory (FMI) [18]. For this study we used the 14-item short version which was found to be semantically robust and psychometrical stable (Cronbach's alpha = .86). The short scale had one common factor and correlated strongly with self-awareness (self-knowledge) [18]. Nevertheless, an alternative two-dimensional solution with the factors ‘‘presence” (alpha = .77) and ‘‘acceptance” (alpha = .69) was tested [19]. For this analysis we used both the FMI sum score (using 14 items) and the two subscales with 4 items each.

### 2.3. Statistical Analyses

All data were treated as ordinal data. The reliability of the scale was evaluated with internal consistency coefficients. Cronbach's coefficient alpha [20] was used to evaluate the reliability of our questionnaire and interitem correlations as published elsewhere [21]. To combine several items with similar content, we relied on the technique of factor analysis (principal component analysis) using Varimax Rotation with Kaiser Normalization. 

Reliability and factor analyses, analyses of variance (ANOVA), and correlation and regression analyses were performed with SPSS 17.0 for Windows (SPSS GmbH Software, Munich). We judged *P* < .05 as significant. With respect to the correlation analyses, *r* > .5 is regarded as a strong correlation, *r* between  .3 and  .5 as a moderate correlation while *r* between  .2 and  .3 is regarded as a weak correlation, and *r* < .2 as no or negligible correlation.

## 3. Results

To validate the questionnaire, we first performed exploratory reliability and factor analyses (study 1) and later on confirmatory analyses (study 2).

### 3.1. Study 1: Exploratory Analyses

#### 3.1.1. Individuals

Attendants of EYT were significantly older than Yoga practitioners, had higher symptom scores, and were practicing much shorter in terms of length of time (Table 1). In fact, EYT was most often applied because of patients' health problems while Yoga was practiced most often by healthy individuals. Thus, most Yoga practitioners were healthy whereas EYT practitioners had health conditions such as cancer, chronic pain, other chronic diseases, mental health affections, and others (Table 1). Most attendants of EYT and Yoga were women, living with a partner and had a higher educational level (Table 1).

#### 3.1.2. Reliability Statistics and Factor Analysis

Item B5 (During the exercises and movements I followed certain “inner pictures” which arose) was regarded as an exclusive maker item for EYT and was thus not enrolled for reliability analysis in the whole sample enrolling also Yoga attendants. Three cognitive items (B2, B11, and B12) had a poor corrected item-total correlation (<.3) and were eliminated from the item pool. However, two of them had a satisfying internal consistency (B2, B12; alpha = .763) and thus were used as a contrasting cognitive scale (*Focus on Perfect Form of Movements*).

As shown in Table 2, the remaining twelve emotional/spiritual/behavioral driven items dealing with the inner consistence with practices and movements had a good internal consistency (alpha = .837). The difficulty index of the twelve items was  .72 (2.88 [mean]/4). Three items (B1, B14, and B15) had a difficulty index >.80, indicating ceiling effects.

Factor analysis revealed a Kaiser-Mayer-Olkin value of  .86, which as a measure for the degree of common variance indicates that the item-pool is suitable for a factorial validation. Exploratory factor analysis pointed to a 3-factor solution which explains 56% of variance (Table 2), that is, one major 7-item scale (*Inner Correspondence with Practices/Easiness) *which had a good internal consistency (alpha = .83) and explains 27% of variance, and a 3-item scale (*Performance with inner involvement*) with a weak internal consistency (alpha = .54) and one item with a factor loading <.5, and a 2-item factor (*Successful transformation into movement) *which had a weak internal consistency, too (alpha = .59).

#### 3.1.3. Correlation Analyses

As shown in Table 3, the scales *Inner Correspondence with Practices/Easiness *and *Performance with inner involvement* were strongly intercorrelated while *Successful transformation into movement* was moderately associated with the aforementioned scales. *Focus on Perfect Form of Movements* correlated just weakly with the *Inner Correspondence with Practices/Easiness *and *Performance with inner involvement* scales.

Weak to moderate associations with life satisfaction dimensions and also with patient satisfaction were found only with respect to *Inner Correspondence with Practices/Easiness* while *Performance with inner involvement* or *Successful transformation into movement *correlated just weakly (or not at all) with the satisfaction scales.

The duration of practice was moderately associated with *Inner Correspondence with Practices/Easiness *but with none of the two minor scales.

#### 3.1.4. Differences between EYT and Yoga Practitioners

There were no significant differences with respect to gender, age, religious denomination, and disease categories (data not shown). *Successful transformation into movement *was the lowest in individuals with low educational level (*F*(3,485) = 3.8; *P* = .010). In contrast *Focus on Perfect Form of Movements *was the lowest in individuals with a high school education (*F*(3,490) = 4.0, *P* = .007). 

The main factor *Inner Correspondence with Practices/Easiness* differed significantly between EYT and yoga practitioners (68.9 ± 16.1 in EYT versus 73.0 ± 14.8 in yoga; *F*(1,498) = 8.5; *P* = .004). In contrast, *Performance with inner involvement* (87.4 ± 14.4 versus 83.0 ± 13.8; *F* = 1.2, n.s.), *Successful transformation into movement *(59.7 ± 23.7 versus 61.1 ± 23.3; *F* = 0.4; n.s.), and *Focus on Perfect Form of Movements* (75.0 ± 22.8 versus 77.1 ± 20.1; *F* = 1.1, n.s.) did not differ significantly between both groups. 

The aforementioned significant differences were due to the items B6 (*F*(1,394) = 27.7; *P* < .0001), B13 (*F*(1,391) = 15.8; *P* < .0001), and B3 (*F*(1,495) = 10.9; *P* = .001), which were of significantly higher relevance in Yoga practitioners. In contrast, the EYT marker item B5 was scored significantly higher in the EYT group (*F*(1,394) = 12.2; *P* = .001) while item B12 from the cognitive scale was higher in the yoga group (*F*(1,393) = 6.2; *P* = .013).

#### 3.1.5. Preliminary Conclusion

Weaknesses/problems of the two minor scales were noted as follows: (1) weak internal consistency, (2) negatively phrased items that require recoding, and (3) weak associations with life satisfaction and health situation (in contrast with duration of practice). Hence, we decided to focus on the main scale *Inner Correspondence with Practices/Easiness *which had a good internal consistency. To improve its quality, we added eight new items (* in Table 4) which refer to the primary intention and performed reliability analysis and a confirmatory factor analysis in smaller sample (study 2).

### 3.2. Study 2: Confirmatory Analyses

The intention of this study was to further validate the main scale of the instrument in a different sample of yoga and EYT practitioners, that is, to perform confirmatory analyses of the extended scale, to approve associations with life and patient satisfaction, and to study its relationship to mindfulness as a potentially associated construct. 

#### 3.2.1. Individuals

For the confirmatory analyses we referred to a sample of 135 individuals (81% women; mean age 51 ± 12 years; 44% married, 17% living with a partner, 20% divorced, 16% single, 2% widowed; 46% high school education, 31% junior high school, 13% secondary school, 11% other; 71% Yoga). In the sample, 25% stated to be healthy, 20% had psychiatric disorders, 26% had other chronic diseases, 7% had cancer, 11% had chronic pain diseases, and 12% had others. The symptom score of the individuals was 35 ± 24, and they were predominantly long-term practitioners (81 ± 98 months).

#### 3.2.2. Reliability and Factor Analyses

Due to a weak corrected item-total correlation, we had to eliminate the items of the former scale 2 *(Performance with inner involvement) *and also from scale 3 *(Successful transformation into movement), *and item B13* (Learned practices were easy for me)* from scale 1* (Learned practices were easy for me)*; item B7 which loaded strongly (.550) on two scales was also excluded. The remaining twelve items had a very good internal consistency (alpha = .948). Principal component analysis (Kaiser-Mayer-Olkin value  .938) confirmed one common factor (eigenvalue 7.7) which explains 64% of variance (Table 4), termed *Inner Correspondence/Peaceful Harmony with Practices* (ICPH). All loadings were between  .73 and  .90.

#### 3.2.3. Correlation and Regression Analyses

As shown in Table 5, the ICPH scale correlated strongly (*r* = .56; *P* < .001) with mindfulness, moderately with life satisfaction (*r* = .38; *P* < .001) and patient satisfaction (*r* between  .23 and  .43; *P* < .01), and negatively with the symptom score (*r* = −.23; *P* = .011). Duration of practice correlated weakly (*r* = .28; *P* = .004) with the scale, but not the duration of disease (*r* = .03; n.s.). Stepwise regression analyses (enrolling variables such as acceptance, presence, age, life satisfaction, patient satisfaction, duration of practice, and symptom scores) confirmed that ICPH can be predicted solely by the acceptance component of mindfulness (Beta = .527; *t* = 5.62, *P* < .0001). The regression model was able to explain 28% of variance (*R* ^2^).

#### 3.2.4. Differences between EYT and Yoga Practitioners

The mean ICPH score of the EYT sample was 63.0 ± 25.8, and 74.8 ± 16.3 in the yoga group. These differences are statistically significant (*F*(1,132) = 9.9; *P* = .002). The ICPH scores did not significantly differ with respect to family status, educational level, and religious denomination (data not shown). However, women had slightly higher scores than men, that is, 73.3 ± 19.3 versus 62.8 ± 22.4 (*F*(1,130) = 5.6; *P* = .019). This effect can be explained by the fact that women had significantly higher mindfulness scores than men (*F*(1,131) = 8.3; *P* = .005), which is the main predictor of ICPH.

## 4. Discussion

Our intention was to develop a scale which operationalizes and measures the “inner involvement” of individuals engaged in specific mind body interventions. A hypothesis was that this active attitude is dependent on the duration of practice and is associated with life satisfaction. One may assume that an inner resistance towards the practices may decrease treatment efficacy, and thus satisfaction with the treatment, health situation, and overall satisfaction with various dimensions of life. Exploratory analyses indicated that the 8-item scale *Inner Correspondence with Practices/Easiness* had a good internal consistency and fits best to the aforementioned hypothesis while the other scales were less satisfying. To improve the quality of the favored scale, we added some new items. Confirmatory reliability and factor analysis with the extended item pool indicated that the new scale *Inner Correspondence/Peaceful Harmony with Practices* (ICPH) has a very good internal consistency (alpha = .948), explained 64% of variance, and correlated strongly with mindfulness (particularly Acceptance), moderately with life satisfaction (particularly with Self) and patient satisfaction (particularly satisfaction with treatment efficacy), and weakly with duration of practice, and (negatively) with the symptom scores. 

The items of this scale address feelings of harmony with the practices in terms of an emotional resonance instead of a cognitive focus on perfect performance. As a result, the individuals feel calm, peaceful, comfortable, and relaxed (nothing from the “outside” can distract them), and they are able to “forget the worries of daily life” while performing the practices; moreover, they are emotionally touched (heart and soul) by the practices. With respect to content validity, the items of the ICPH scale are sound and well suited. 

One may also assume construct validity (which is underlined by a high internal consistency coefficient) because the ICPH scores were significantly higher in the yoga practitioners which were practicing much longer (104 ± 100 months) than the EYT practitioners (3 ± 6 months) and were dependent on life and patient satisfaction, mindfulness and weakly on duration of practice and symptom score. However, a limitation of the study is that we have no data on health-related quality of life as comeasurements, which will be addressed in future longitudinal studies.

The strong correlation of the ICPH scale with mindfulness is of conceptual importance. The acceptance component of mindfulness construct is related to the nonjudgmental acceptance of the situation while mindfulness presence is related to the experience of the moment and a cognitive reflection of all actions [18, 19]. Thus, the inner correspondence and feelings of peaceful easiness with the practices is related (in terms of the FMI items) particularly with the feeling to be connected to the “experience in the here-and-now”, to the ability to “appreciate myself”, to be “friendly when things go wrong”, to “pause without immediately reacting” even in difficult situations, and the experience of “moments of peace and ease” even in stressful situations. In this study, the acceptance component of mindfulness correlated with the duration of practice (*r* = .50; *P* < .001), negatively with the symptom scores (*r* = −.41; *P* < .001) and strongly with life satisfaction (*r* = .60; *P* < .001). In fact, stepwise regression analyses confirmed that ICPH can be predicted solely by the acceptance component of mindfulness. Because mindfulness seemed to be predictive of lower psychological distress [18], it remains to be investigated whether mental health is associated with ICPH too. 

**Figure 1:**
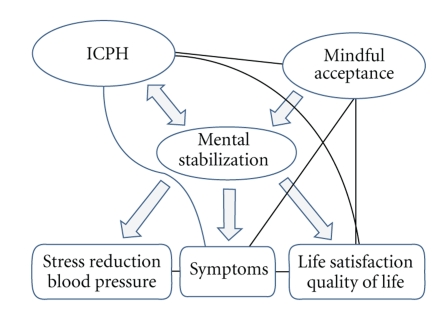
Hypothetical model of the interconnections between ICPH, mindful acceptance and mental/emotional stabilization in response to mind-body interventions such as yoga or EYT.

One could suggest that if distinct mind-body interventions such as EYT and yoga are associated with psychical stabilization and consecutive stress-reducing effects, this could be mediated (at least in part) by an increase of mindful acceptance (Figure 1). Our scale ICPH seems to measure a unique facet of this mental stabilization. Findings of Shapiro et al. [13] that yoga class completers with major depression in remission had a greater capacity for emotional regulation as compared to the nonremitters underline the hypothesis that the emotional stabilization, mindfulness, and inner congruence might be interconnected. 

Nevertheless, one cannot ignore the fact that also positive expectations of the patients are modulating factors contributing to the clinical effects of mind-body interventions influencing behavior which in turn can have a strong impact on health. Expectations are thought to underlie so-called “placebo effects”, impacting perceptions and biological processes. One should realize that the “placebo effect” itself represents a true measurable correlate of an organism's psycho-neurobiological response and, thereby, influences the healing processes [22]. The “placebo effect” is taken to mean also the broad array of nonspecific effects in the patient-physician relationship, including attention, compassionate care, and the modulation of expectations, anxiety, and self-awareness [23]. Thus, if one intends to analyze the clinical effects of distinct interventions of mind-body-medicine, one has to be aware of a complex interacting system enrolling patients' expectations, inner engagement, life satisfaction, mental stabilization, attention by the therapist, and also physical effects of the interventions. For confirmatory clinical studies on EYT, yoga and so forth, these issues need to be addressed. 

## 5. Conclusion

The 12-item scale *Inner Correspondence/Peaceful Harmony with Practices* (ICPH) is a useful, valid, and reliable instrument to assess the impact of individuals' inner correspondence and feelings of peaceful easiness with mind-body interventions. A limitation of the study is the lack of scale stability investigations (i.e., retest reliability). To address this and to investigate the scale's “sensitivity to change” in response to interventions, we are currently running longitudinal studies enrolling yoga and EYT practitioners. These studies address additional co-measures such as health-related quality of life, mood states, and aspects of spirituality. One may also assume state- and trait-like components of the ICPH which remain to be clarified. The instrument can be used in clinical studies to assess the efficacy of mind body practices involving physical movements; its suitability to assess the effects of more static meditative mind body interventions remains to be investigated.

## Figures and Tables

**Table 1:** Demographic data of individuals performing EYT or Yoga.

	EYT	Yoga	Significance
Age (years)	51.5 ± 13.2	44.7 ± 11.1	<.001*

Gender (%)			.010**
Women	81	90	
Men	19	10	

Family status (%)			.001**
Married	46	53	
Living with partner	10	19	
Divorced	11	11	
Living alone	28	16	
Widowed	6	2	

Educational level (%)			.004**
Secondary	9	7	
Junior high school	23	27	
High school	49	58	
Other	19	8	

Religious denomination			.024**
Christian	77	69	
Others	6	6	
None	18	26	

Duration of practice (month)	21.8 ± 50.7	72.8 ± 84.1	<.001*

Disease categories (%)			<.001**
Healthy	2	46	
Cancer	13	4	
Chronic pain diseases	8	10	
Other chronic diseases	37	18	
Psychic disorders	26	11	
Other affections	14	11	

Symptom Score (VAS)	54.3 ± 18.8	27.3 ± 23.4	<.001*

*ANOVA; **Pearson's Chi^2^ test (2-tailed).

**Table 2:** Items, factor loading, internal consistencies, and mean values of EYT and Yoga groups (study 1).

Items	Factor loading			Mean	SD	Difficulty index
	1	2	3			
*Inner Correspondence with Practices/ Easiness* (eigenvalue 4.5; alpha = .83)						
B3 during the practices there was no need to think about it; I could fully get into it	**.756**			2.65	1.04	0.66
B6 during the practices I was able to forget myself completely	**.695**			2.35	1.16	0.59
B13 learned practices were easy for me	**.683**			2.35	0.98	0.70
B4 Emotionally, I could completely come to terms with the practices	**.671**	.313		3.01	0.86	0.75
B9 feelings and intentions corresponded with movements	**.641**	.325	.360	2.80	0.93	0.70
B16 felt comfortable and relaxed during my practices	**.550**			2.96	0.89	0.74
B7 full heartedly within the practices	**.550**	.500		3.14	0.79	0.59

*Performance with inner involvement *(eigenvalue 1.2; alpha = .54)						
B15 mostly performed practices without real inner involvement (-)		**.795**		3.42	0.83	**0.86**
B14 performed the learned practices with much inner resistance (-)		**.624**	.350	3.41	0.87	**0.85**
B1 performed the learned practices consciously	.445	.493		3.57	0.58	**0.89**

*Successful transformation into movement* (eigenvalue 1.0; alpha = .59)						
B10 did not really succeed in transforming my “inner pictures” (images in my mind) into movements (-)			**.762**	2.31	1.05	0.58
B8 not really successful in balancing movements with feelings and intentions (-)			**.757**	2.55	1.16	0.58

*Focus on Perfect Form of Movements *(alpha = .76)						
B2 tried to perform the learned practices perfectly				3.18	088	0.80
B12 While performing the practices, I am fully focused on their perfect accomplishment				2.93	1.00	0.73

*B5 During the exercises and movements I followed certain “inner pictures” which arose.				2.17	1.23	0.54

Principal component analysis; Varimax rotation with Kaiser normalization (rotation converged in 8 iterations). (-) recoded items.

*marker item specific for EYT.

**Table 3:** Correlation analyses (study 1).

	Inner Correspondence with Practices/Easiness	Performance with inner involvement	Successful transformation into movement
Inner correspondence with practices/easiness	1.000	.514**	.378**
Performance with inner involvement		1.000	.346**
Successful transformation into movement			1.000

Focus on Perfect Form of Movements	.262**	.206**	.021

Life Satisfaction			
BMLSS Sum Score	.373**	.224**	.146**
Family life	.249**	.213**	.150**
Friendships	.299**	.232**	.140**
Work	.311**	.164**	.121
Self	.374**	.180**	.125**
Where I live	.238**	.194**	.056
Overall life	.346**	.218**	.155**
Financial situation	.128**	.103	.045
Future perspectives	.306**	.188**	.137**

Patient satisfaction			
Health situation	.315**	.146**	.168**
Treatment efficacy	.381**	.233**	.227**
Ability to manage daily life	.348**	.218**	.150**

Symptoms (VAS)	−.118	.006	−.121**

Duration of practice	.305**	.050	.099

**P < .01 (Spearman rho; 2-tailed).

**Table 4:** Items, internal consistencies, and factor loading of the extended scale 1 (study 2).

Inner correspondence/peaceful harmony with practices (ICPH) (eigenvalue 7.7; alpha = .948)	Corrected item-total correlation	Cronbach's alpha if item deleted	Factor loading
B21* entire sensation was in harmony with the movements/practices	.871	.940	**.897**
B18* felt in complete harmony with the practices deep within	.809	.942	**.845**
B9 feelings and intentions corresponded with movements	.790	.942	**.828**
B6 during the practices I was able to forget myself completely	.772	.943	**.814**
B4 emotionally, I could completely come to terms with the practices	.776	.943	**.814**
B23* calm and peaceful while performing the practices	.772	.943	**.813**
B22* able to forget the worries of daily life while performing the practices	.729	.944	**.779**
B20* practices have deeply touched and warmed my heart and soul	.724	.944	**.773**
B24* performing the practices, completely engrossed—nothing from the outside distracts	.725	.944	**.772**
B17* easy to have a sense of my “inner pictures” (images in my mind) and to bring them to life within	.724	.945	**.771**
B3 during the practices there was no need to think about it; I could fully get into it	.713	.945	**.760**
B16 felt comfortable and relaxed during my practices	.681	.946	**.732**

Principal component analysis; one extracted component which explains 64% of variance.

*new items.

**Table 5:** Correlation of ICPH with other constructs (study 2).

	Inner Correspondence/Peaceful Harmony (ICPH)
Mindfulness	
Sum score	.563**
Mindful presence	.416**
Nonjudgmental acceptance	.536**

Life Satisfaction	
BMLSS Sum Score	.379**
Family life	.203
Friendships	.347**
Work	.402**
Self	.424**
Where I live	.191
Overall life	.396**
Financial situation	.147
Future perspectives	.334**

Patient satisfaction	
Health situation	.345**
Treatment efficacy	.427**
Ability to manage daily life	.327**

Symptoms (VAS)	−.231**

Duration of practice (months)	.280**

Duration of disease (months)	.029

**P < .01 (Spearman rho; 2-tailed); strong correlations were highlighted.
